# Diagnostic contribution of metabolic workup for neonatal inherited metabolic disorders in the absence of expanded newborn screening

**DOI:** 10.1038/s41598-019-50518-0

**Published:** 2019-10-01

**Authors:** Alexandra Bower, Apolline Imbard, Jean-François Benoist, Samia Pichard, Odile Rigal, Olivier Baud, Manuel Schiff

**Affiliations:** 1 Neonatal intensive care department, Robert Debré University Hospital, APHP, Paris, 75019 France; 2 Reference Center for Inborn Errors of Metabolism, Robert Debré University Hospital, APHP, Paris, 75019 France; 3 Biochemistry Laboratory, Robert Debré University Hospital, APHP, Paris, France; 4 Paris Sud University, Chatenay Malabry, France; 5 UMR1141, PROTECT, INSERM, Université de Paris, Paris, 75019 France

**Keywords:** Metabolic disorders, Paediatric research

## Abstract

Inherited metabolic disorders (IMDs) in neonates are a diagnostic and therapeutic challenge for the neonatologist, with the priority being to rapidly flag the treatable diseases. The objective of this study was to evaluate the contribution of targeted metabolic testing for diagnosing suspected IMDs on the basis of suggestive clinical setting or family history in neonates. We conducted an observational study over five years, from January 1st, 2010 to December 31, 2014 in the neonatal intensive care unit (NICU) at Robert Debré University Hospital, Paris, France. We assessed the number of neonates for whom a metabolic testing was performed, the indication for each metabolic test and the diagnostic yield of this selected metabolic workup for diagnosing an IMD. Metabolic testing comprised at least one of the following testings: plasma, urine or cerebrospinal fluid amino acids, urine organic acids, plasma acylcarnitine profile, and urine mucopolysaccharides and oligosaccharides. 11,301 neonates were admitted at the neonatal ICU during the study period. One hundred and ninety six neonates underwent metabolic testing. Eleven cases of IMDs were diagnosed. This diagnostic approach allowed the diagnosis, treatment and survival of 4 neonates (maple syrup urine disease, propionic acidemia, carnitine-acylcarnitine translocase deficiency and type 1 tyrosinemia). In total, metabolic testing was performed for 1.7% of the total number of neonates admitted in the NICU over the study period. These included 23% finally unaffected neonates with transient abnormalities, 5.6% neonates suffering from an identified IMD, 45.4% neonates suffering from a non-metabolic identified disease and 26% neonates with chronic abnormalities but for whom no final causal diagnosis could be made. In conclusion, as expected, such a metabolic targeted workup allowed the diagnosis of classical neonatal onset IMDs in symptomatic newborns. However, this workup remained normal or unspecific for 94.4% of the tested patients. It allowed excluding an IMD in 68.4% of the tested neonates. In spite of the high rate of normal results, such a strategy seems acceptable due to the severity of the symptoms and the need for immediate treatment when available in neonatal IMDs. However, its cost-effectiveness remains low especially in a clinically targeted population in a country where newborn screening is still unavailable for IMDs except for phenylketonuria in 2019.

## Introduction

Inherited metabolic diseases (IMD) represent roughly over 700 different genetic disorders. They occur especially during infancy and are particularly severe in the neonatal period. Though the individual incidence is low (1 for 10,000 to 1 for 1 million), their collective incidence is high^1,2^. Despite the rarity of these diseases, making a rapid diagnosis of an IMD is a challenge for the neonatologist, considering the severity of the clinical presentation and some available treatments that have to be urgently initiated^3^.

A growing number of patients with IMD are identified pre-symptomatically by population neonatal screening (NBS) programmes^4,5^. However, some neonates escape early detection because their symptoms and signs start before NBS results become available, they sometimes even die before sample for NBS has been drawn or because there are IMDs which are not included in the NBS programmes^6^. In 2019 in France, the sole IMD currently covered by NBS is phenylketonuria (PKU)^7^. Expanding NBS to other IMDs is currently under evaluation by public health authorities with the hope to implement expanded NBS in the near future.

IMDs are generally divided into three pathophysiological categories: disorders that give rise to intoxication, disorders involving energy metabolism and disorders involving complex molecules^8–10^. The diagnostic approach relies on nonspecific clinical and laboratory testing data, leading to the prescription of metabolic tests^6–11^. Starting a treatment is an emergency and should be done without waiting for the results. Final confirmation of the IMD relies on molecular testing^4,12^. The absence of specificity of clinical signs in neonates and the numerous nonspecific abnormalities of metabolic testing make the diagnosis of IMD difficult in the neonatal period^11,13–16^. As for any “neonatal” disease, for example infection or anoxia, symptoms include poor sucking, lethargy, hypotonia, and respiratory distress. IMDs should be suspected when no specific aetiology is found and symptoms persist despite appropriate management^17^. Initial laboratory parameters can suggest the diagnosis of IMD when revealing for instance, metabolic acidosis, high ammonia, refractory hypoglycaemia and liver failure^8^. If the clinical and laboratory suspicion is strong, the second step is to order metabolic testing. Identification of metabolites is essential, as it allows, from easily accessible biological samples (blood, urine, sometimes cerebrospinal fluid [CSF]), to rapidly guide the diagnosis^18,19^. Narrow collaboration between clinicians and biochemists is essential for interpreting those tests (clinical data, condition of sample collection, transport) as many exogenous factors may interfere with the interpretation of the results of metabolic testing. Plasma amino acids results may be influenced by nutritional status, independently of age^20^. For example, lack of enteral feeding can lower citrulline concentrations. Deficiency in vitamin B1 leads to rising of proline and alanine. In neonates with total parenteral nutrition (TPN), deficiency in tyrosine is possible. Some drugs may lead to metabolic disturbance, either by modifying the concentration of a given amino acid or because they contain some. For example, sodium valproate raises glycine concentration and disturbs urinary organic acids and acylcarnitines profile. Gestational age has to be taken into account too, as renal and liver immaturity can modify results^13^.

Systematic evaluations of the yield of targeted metabolic testings performed in symptomatic neonates in countries without expanded NBS are scarce^21^. Our objective was therefore to retrospectively evaluate the diagnostic contribution of selected metabolic tests (covering most of the treatable IMDs and performed in a single metabolic laboratory) when an IMD is suspected in the neonatal period.

## Methods

### Patients

We conducted an observational study of neonates admitted in the neonatal intensive care unit (NICU) in Robert Debré University Hospital (Paris, France) from January 1, 2010 to December 31, 2014. The total number of admitted neonates over this study period was 11,301. Neonates were included if they were suspected of IMD based on clinical suggestive setting (Table 1) and/or family history of IMD and had undergone at least one of the following metabolic testings: plasma, urine or cerebrospinal fluid (CSF) amino acids, urine organic acids, plasma acylcarnitine profile, and urine mucopolysaccharides and oligosaccharides.

**Table 1:** General indication for each metabolic test.

Category of laboratory parameter	Clinical signs and symptoms indication
p/u/CSF AA	Acute neurological deterioration
	High ammonia
	Liver failure
uOA	Acute neurological deterioration
	Liver failure
	Metabolic acidosis (ketoacidosis, lactic acidosis)
	High ammonia
Plasma acylcarnitines	Acute neurological deterioration
	Liver failure
	High ammonia
	Metabolic acidosis (lactic acidosis)
	Heart failure
	Cardiomyopathy
	Hypoketotic hypoglycemia
	High CPK
MPS/OS	Organomegaly (liver, spleen)
	Hydrops fetalis
	Abnormal neurological status in the context of organomegaly

Indications for each metabolic testing.

pAA: plasma aminoacid chromatography; uAA: urine aminoacid chromatography; CSFAA: cerebrospinal fluid aminoacid chromatography; uOA: urine organic acid chromatography; ACYL: acylcarnitine profile; OS: urine oligosaccharides; MPS: urine mucopoysaccharides.

The ethics committee of Robert Debré University Hospital (APHP, 75019 Paris, France) approved the study. All procedures conformed to ethical standards. Informed consent was obtained from parents.

### Data collection

Data were collected retrospectively through the in-house database of the Biochemistry laboratory of the hospital. The clinical signs, laboratory results, functional tests and available imaging results that were part of the diagnostic approach of neonatal IMD suspicion were collected. The clinical data collected were: consanguinity, family history of IMD or unexplained neonatal death; clinical signs appearing after a symptom-free period; neurological signs (neurological deterioration with altered consciousness [from lethargy to coma], axial hypotonia, peripheral hypertonia, seizures); hepatomegaly; definitive diagnosis if available.

Data on the initial laboratory tests collected were: persisting hyperlactacidemia (blood lactate >2.5 mmol/L persisting over 6 hours of life). Indeed, hyperlactacidemia due to perpartum asphyxia should normalize within 6 hours^22^; hypoglycemia <2.8 mmol/L persisting after glucose administration (<2.5 mmol/L within the 24 first hours); blood ammonia >100 µmol/L; liver failure, defined by prothrombin time (PT) ratio and factor V < 50%^23,24^.

We collected data on electroencephalograms (EEG) and cerebral magnetic resonance imaging (MRI) for neurological presentations, and echocardiography and electrocardiogram for cardiac presentations^25^.

For differential diagnosis with neonatal hypoxia, the Apgar score was collected since an Apgar score less than 7 at 5 minutes is correlated with increased risk of encephalopathy^22^.

### Patients groups

Neonates who had a metabolic testing were divided into 4 groups according to the definitive diagnosis/conclusions at the end of the hospitalization in the NICU: group 0, neonates with transient abnormalities; group 1, neonates diagnosed with an IMD genetically confirmed; group 2, neonates suffering from a non-metabolic identified disease (differential diagnosis); group 3, neonates with chronic disorders but for whom no final diagnosis could be made.

Definitive confirmation of IMD was made by molecular testing including segregation of the mutations found by sequencing DNA from the parents.

### Classification of metabolic testing results

Metabolic analyses were classified according to their results into 3 categories: normal result, specific abnormality pointing towards a specific IMD, nonspecific abnormalities including those due to an unequivocal exogenous factor confirmed by the biochemistry laboratory (e.g: nutrition containing medium chain triglycerides, drugs…). Among the nonspecific abnormalities, we reviewed the analyses that were re-performed.

## Results

**Figure 1:**
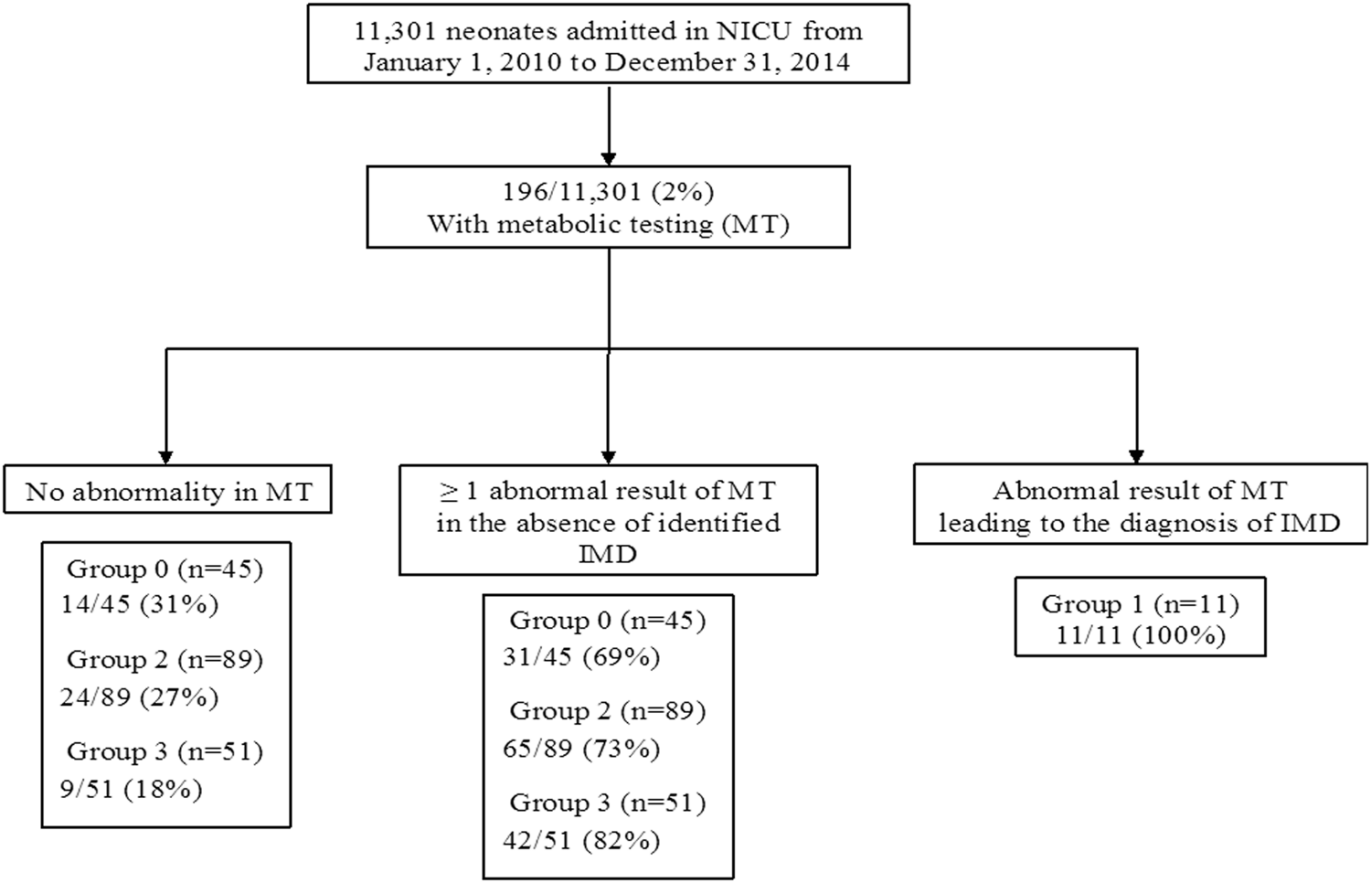
Flow chart of the study.

Among the 11,301 admitted neonates, 196 had at least one of the above detailed metabolic testings for suspected IMD (Fig. 1). The demographic characteristics of those 196 patients are presented in Table 2.

**Table 2:** Demographic characteristics of the studied population of 196 neonates for whom metabolic testings were performed.

	Mean ± SD (min-max)
	or n/N (%)
**Sex**	
Male	104/196 (53%)
Female	93/196 (47%)
Gestationnal age (*weeks*)	36.7 ± 7.1 (25–42)
Birth weight (*g*)	2624.5 ± 947 (650–4400)
Age at admission (*days*)	5.7 ± 11.9 (0–60)

**Figure 2:**
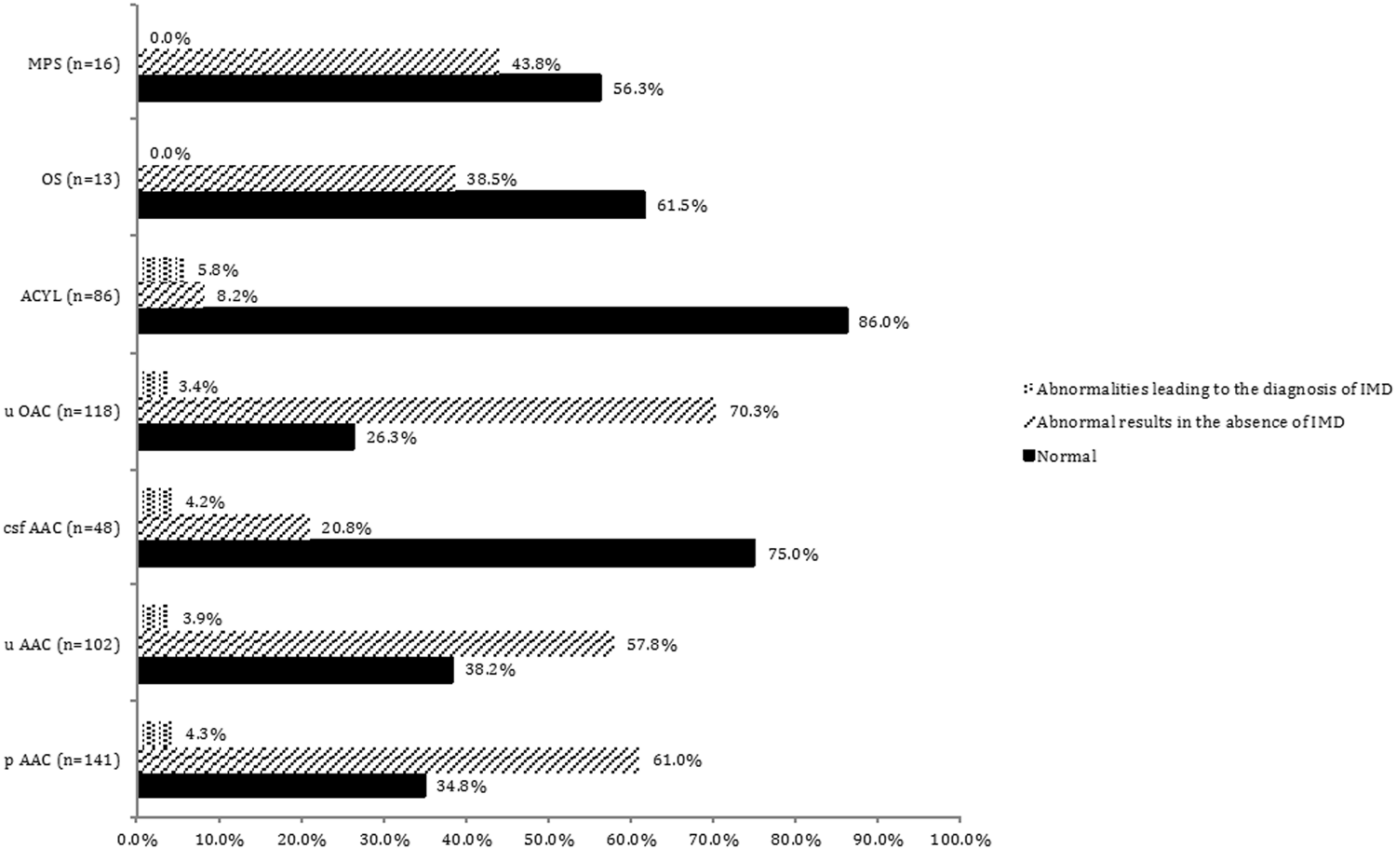
Results of metabolic testings in the whole population (N = 196). pAA: plasma aminoacid chromatography; uAA: urine aminoacid chromatography; CSFAA: cerebrospinal fluid aminoacid chromatography; uOA: urine organic acid chromatography; ACYL: acylcarnitine profile; OS: urine oligosaccharides; MPS: urine mucopoysaccharides.

Among metabolic testings in our population, abnormalities were specific of an IMD for 4.3% of plasma aminoacids, 3.9% of urine aminoacids, 4.2% of CSF aminoacids, 3.4% of urine organic acids, 5.8% of plasma acylcarnitine profile (Fig. 2).

**Figure 3:**
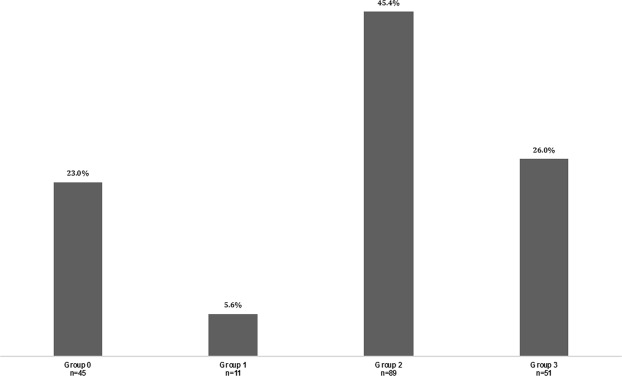
Classification of patients into groups. Group 0: neonates with transient disorder; group 1: neonates diagnosed with IMD confirmed by molecular testing; group 2: neonates suffering from a non-metabolic disease; group 3: neonates with chronic clinical abnormalities but for whom no diagnosis could be made.

There were 23% neonates with a final diagnosis of transient disorder (group 0), 5.6% neonates diagnosed with an IMD (group 1) confirmed by molecular testing [a) 4 aminoacidopathies: 1 case of maple syrup urine disease (MSUD), 1 hereditary tryosinemia type 1 (HT1), 2 nonketotic hyperglycinemia (NKH); b) 2 fatty acid oxidation disorders (FAO): 1 carnitine acylcarnitine translocase deficiency (CACT), 1 Long chain 3-hydroxyacylCoA dehydrogenase deficiency (LCHADD); c) 1 organic aciduria: 1 case of propionic academia (PA); d) 2 mitochondriopathies; e) 2 urea cycle disorders: 1 N-acetylglutamate synthase deficiency (NAGS), 1 citrullinemia type 1], 45.4% neonates suffering from a non-metabolic identified disease (group 2, Supplemental Table 1) and 26% neonates with chronic clinical abnormalities but for whom no final diagnosis could be made (Fig. 3).

Patients’ clinical and laboratory results are presented in Tables 3 and 4 respectively, by group category. Results of brain imaging/EEG/heart involvement and Apgar score by group category are presented in Tables 5 and 6 respectively.

**Table 3:** Clinical characteristics in the 196 neonates for whom metabolic testings were performed.

		Group 0	Group 1	Group 2	Group 3
		N = 45	N = 11	N = 89	N = 51
		n (n/N)	n (n/N)	n (n/N)	n (n/N)
Medical history	Consanguinity	6 (13.3%)	3 (27.3%)	4 (4.5%)	5 (9.8%)
	Family history of IMD or unexplained death in neonatal period	7 (15.6%)	9 (81.8%)	8 (9%)	12 (23.5%)
	Symptom-free period	4 (8.9%)	5 (41.7%)	7 (7.9%)	6 (11.8%)
Neurological signs	Neurological deterioration	0 (0%)	6 (54.5%)	37 (41.6%)	19 (37.2%)
	Axial hypotonia	7 (15.6%)	9 (75%)	40 (44.9%)	36 (70.6%)
	Peripheral hypertonia	1 (2.2%)	2 (16.7%)	0 (0%)	5 (9.8%)
	Clinical seizures	6	5 (41.7%)	24 (27%)	12 (23.5%)
Liver signs	Hepatomegaly	0 (0%)	3 (25%)	32 (36%)	19 (37.2%)

Group 0: neonates with a final diagnosis of transient disorder; group 1: neonates diagnosed with IMD confirmed by molecular testing; group 2: neonates suffering from a non-metabolic disease; group 3: neonates with chronic clinical abnormalities but for whom no diagnosis could be made.

**Table 4:** Laboratory parameters data in the 196 neonates for whom metabolic testings were performed.

	Group 0	Group 1	Group 2	Group 3
	N = 45	N = 11	N = 89	N = 51
	n (n/N)	n (n/N)	n (n/N)	n (n/N)
Persistent hyperlactacidemia >6 h	2 (4.4%)	4 (33.3%)	1(1.1%)	6 (11.8%)
Hypoglycemia	15 (33.3%)	2 (16.7%)	8 (9.0%)	5 (9.8%)
High ammonia (>100 µmol/L)	0 (0%)	5 (41.7%)	3 (3.4%)	1 (2.0%)
Liver failure (PT, FV)	1 (2.2%)	2(16.7%)	10 (11.2%)	3 (5.9%)

Group 0: neonates with a final diagnosis of transient disorder; group 1: neonates diagnosed with IMD confirmed by molecular testing; group 2: neonates suffering from a non-metabolic disease; group 3: neonates with chronic clinical abnormalities but for whom no diagnosis could be made.

**Table 5:** Brain imaging (MRI), electroencephalogram (EEG) and heart findings in the 196 neonates for whom metabolic testings were performed.

	Group 0	Group 1	Group 2	Group 3
	N = 45	N = 11	N = 89	N = 51
Abnormal brain MRI	2 (4.4%)	3 (27.3%)	35 (39.3%)	29 (57%)
Abnormal EEG	0 (0%)	3 (25%)	32 (36%)	19 (37%)
Cardiomyopathy	0 (0%)	1 (8.3%)	6 (6.7%)	0 (0%)

Group 0: neonates with a final diagnosis of transient disorder; group 1: neonates diagnosed with IMD confirmed by molecular testing; group 2: neonates suffering from a non-metabolic disease; group 3: neonates with chronic clinical abnormalities but for whom no diagnosis could be made.

**Table 6:** Apgar score in the 196 neonates for whom metabolic testings were performed.

	Group 0	Group 1	Group 2	Group 3
	N = 45	N = 11	N = 89	N = 51
	n (n/N)	n (n/N)	n (n/N)	n (n/N)
Apgar <7 at 5 min	5 (11.1%)	0 (0%)	35 (39.3%)	11 (21.6%)

Group 0: neonates with a final diagnosis of transient disorder; group 1: neonates diagnosed with IMD confirmed by molecular testing; group 2: neonates suffering from a non-metabolic disease; group 3: neonates with chronic clinical abnormalities but for whom no diagnosis could be made.

If metabolic testing led to non-diagnostic abnormalities considered as nonspecific, the tests could be re-performed in 56 patients, with normalization (in 37 patients) or persisting abnormalities (in 19 patients), for those patients still suspected of an IMD at the time when the test was re-performed (Table 7). Among the 11 cases of molecularly confirmed IMD, 4 survived. Clinical presentation and initial laboratory parameters of cases of IMD are presented in Tables 8,9 presents results of metabolic testings and molecular findings in these IMD patients. The outcome over a 2 year-period from January 2015 to December 2017 showed that no case of IMD was further diagnosed among patients in groups 0, 2 or 3.

**Table 7:** Nonspecific metabolic abnormalities.

	p AA	u AA	CSF AA	u OA	Acyl	OS	MPS
	N = 86	N = 59	N = 10	N = 83	N = 7	N = 5	N = 7
	n (n/N)	n (n/N)	n (n/N)	n (n/N)	n (n/N)	n (n/N)	n (n/N)
Reperformed analysis	31 (36%)	10 (16.9%)	0 (0%)	10 (12%)	2 (28.6%)	1 (20%)	2 (28.6%)
*Normalisation*	24	4	0	4	2	1	2
*Persisting non specific abnormality*	7	6	0	6	0	0	0

pAA: plasma aminoacid chromatography; uAA: urine aminoacid chromatography; CSFAA: cerebrospinal fluid aminoacid chromatography; uOA: urine organic acid chromatography; ACYL: acylcarnitine profile; OS: urine oligosaccharides; MPS: urine mucopoysaccharides.

**Table 8:** Clinical presentation and initial laboratory parameters of IMD patients.

IMD	Clinical presentation	Laboratory tests abnormalities
***Fatty acid oxidation disorder*** (***FAO***)		
*Translocase deficiency* (*CACT*)	Neurological deterioration at D2, seizures and hypotonia	High ammonia (maximum 1142 µmol/L at H57), no hypoglycemia, normal lactate, no liver failure, normal CPK
*Long chain 3-hydroxyacyl-CoA dehydrogenase deficiency* (*LCHAD*)	Cardiogenic shock	Hyperlactacidemia 14 mmol/L at H36, hypoglycemia, normal ammonia, no liver failure
***Urea cycle disorders***		
*N-acetylglutamate synthase deficiency* (*NAGS*)	Neurological deterioration with symptom free interval of 2 days, seizures,hypotonia and coma	Hyperlactacidemia at 6 mmol/L, High ammonia at 945 μmol/L at H48, no liver failure, no hypoglycemia
*Type 1 citrullinemia* (*ASS*)	Neurological deterioration with symptom free interval of 2 days, hypotonia, seizures and trismus	High ammonia at 787 μmol/L at D 3, liver failure (PT 31%, FV 47%), no hypoglycemia, hyperlactatacidemia
***Aminoacidopathy***		
*Non ketotic hyperglycinemia*	Global hypotonia, no sucking reflex	High ammonia at 109 μmol/L, when reperformed 34 μmol/L; no liver failure, no hypoglycemia nor hyperlactacidemia
*Non ketotic hyperglycinemia*	Global hypotonia, no sucking reflex	No abnormality in initial laboratory tests
*Type 1 tyrosinemia*	Presymptomatic tests for intrafamilial screening (mother with type 1 tyrosinemia)	No abnormality in initial laboratory tests
*Maple syrup urine disease* (*MSUD*)	Neurological deterioration and hypotonia with symptom free interval	No abnormality in initial laboratory tests
***Organic aciduria***		
*Propionic acidemia* (*PA*)	Vomiting	Hyperammoniemia at 111 μmol/L at D18; no liver failure, no hyperlactacidemia nor hypoglycemia
***Mitochondriopathy***		
*DGUOK deficiency*	Presymptomatic testing for a previous case of liver failure which occurred for a sister at 4 months of age	No abnormality in initial laboratory tests
*QIL1 deficiency*	Respiratory failure	Hyperlactacidemia (22.7 mmol/L at D1) and hypoglycemia (1.9 mmol/L at D1), liver failure (PT 29%, FV 30%) NH3 not performed

Clinical presentation and initial laboratory parameters of the identified cases of IMD.

NP: not performed.

**Table 9:** Results of metabolic testings and molecular results of the IMD patients.

IMD	Age at admission	p AA	u AA	CSF AA	u OA	Acyl	OS	MPS	Molecular	Death
***Fatty acid oxidation disorder*** (***FAO***)										
*Translocase deficiency* (*CACT*)	7	Discretely hypoaminoacid profile non suggestive of an aminoacidopathy	Non specific hyperaminoaciduria probably due to renal immaturity	NP	Elevation of dicarboxylic acids, presence of undetermined peaks, probably due to medication	Profile showing elevation of all chain length acylcarnitines	NP	NP	Homozygote mutation c.110 G > C in *SLC25A20* coding for carnitine acylcarnitine translocase	No
*Long chain 3-hydroxyacyl-CoA dehydrogenase deficiency* (*LCHAD*)*:* TFP deficiency	2	High elevation of alanine and proline suggesting hyperlactacidemia	Normal	NP	Elevation of lactate	Elevation of long chain 3-hydroxyacylcarnitines	NP	NP	Homozygote deletion-insertion: c.443-20_624delinsCACACAAG in *HADHB*	neonat.
***Urea cycle disorders***										
*N-acetylglutamate synthase deficiency* (*NAGS*)	0	Elevation of Glu + Gln and lysine, due to hyperammoniemia	Normal	NP	Elevation of lactate, presence of ketone bodies, lack of orotic acid, very discrete elevation of ethylmalonic acid	NP	NP	NP	Homozygote mutation c.1228 T > C in *NAGS* coding for N-acetylglutamate synthase	neonat.
*Type 1 citrullinemia* (*ASS*)	3	Massive elevation of citrulline	NP	NP	NP	NP	NP	NP	Homozygote mutation c.836 G > A in *ASS1* coding for argininosuccinate synthase	neonat.
***Aminoacidopathy***										
*Maple syrup urine disease* (MSUD)	14	Massive elevation of leucine, isoleucine and valine. Presence of alloisoleucine.	massive leucine, isoleucine and valine excretion	NP	Presence of numerous alphaketoacids including alphaKIC	NP	NP	NP	Homozygote mutation p.Gln177X in gene *BCKDHA* coding for alpha unit of the complexe of ramified ceto-acids deshydrogenase	No
*Non ketotic hyperglycinemia*	3	Elevated glycine	Massive hyperglycinuria	Elevated glycine	Numerous interfering peaks probably due to medication	Normal	Normal	NP	Homozygote mutation c.14dup in *AMT*, coding for the unit T (aminomethyltransferase) of GCS	neonat,
*Non ketotic hyperglycinemia*	4	Elevation of glycine; ratio CSF glycine/plasma glycine = 0.05	Massive hyperglycinuria	Isolated elevation of glycine	Isolated elevation of lactate	Normal	NP	NP	p.Gln2X/p.Arg296Pro in *AMT* coding for the unit T of GCS	neonat.
*Type 1 tyrosinemia*	0	Hypertyrosinemia	Rise of 4OH-phénylacetic and pyruvic acids	NP	succinylacetone	NP	NP	NP	Mutation c.554-1 G > T in intron 6 of *FAH* coding for fumarylacetoacetate hydrolase	No
***Organic aciduria***										
*Propionic acidemia*	12	Very high elevation in glycine and lysine	NP	NP	Abnormal profile, elevation in 3OH-propionic and methylcitric acids	elevation of propionylcarnitine	NP	NP	Homozygmutation c.1198 + 1 G > T in *PCCB* coding for beta unit of PCC	No
***Mitochondriopathy***										
*DGUOK deficiency*	0	Elevation in glutamine, tyrosine and phenylalanine	Discrete elevation in tyrosine: liver failure?	NP	Moderate non specific hyperaminoaciduria which can be physiological for the age	Isolated elevation of lactate, to control	NP	NP	Homozygote mutation c.493 G > A in *DGUOK* coding for DGUOK	9mo
*QIL1 deficiency*	0	High elevation in alanine and proline due to hyperlactacidemia	Numerous interfering peaks probably due to medication	NP	Very high elevation of lactate, ketone and dicarboxylic acids	NP	NP	NP	Deletion C.30-1 G > A in exon 2 of *C19ORF70* coding for protein QIL1	2 y

Results of metabolic and molecular testings in these IMD patients.

NP: not performed.

## Discussion

### Diagnostic yield of selected metabolic testing

In terms of epidemiology, the diagnostic yield of metabolic testing seems relatively accurate regarding those IMDs, which are symptomatic in the neonatal period, in a country not using NBS for IMDs other than PKU. Indeed, no patient classified in group 0, 2 or 3 from our population was diagnosed with an IMD within the 2 years period following the study. Moreover, symptoms revealed within the first days of life, which most of the time, is a too short time lapse to obtain the results of NBS. Metabolic testing of at-risk newborns allows a quick diagnosis in the neonatal period before the availability of NBS results.

The clinical features that led to order metabolic testing allowed not to miss any case of IMD, but are not specific. Patients diagnosed with a non-IMD disease could exhibit similar symptoms than those diagnosed with IMD. It seems therefore difficult to select specific (if any) clinical signs to order metabolic tests.

Metabolic testings were performed especially in term neonates (mean gestational age of 36.7 weeks’ gestation). Indeed, most of the IMD neonates are born at term. In the patients who had a metabolic workup, the main clinical presentation was that of a neurological deterioration. The association of a specific medical history and neurological deterioration with symptom-free interval was the most frequent presentation among intoxication-type cases of confirmed IMD (group 1, MSUD and PA). The lack of this association reduced the risk that neurological symptoms were ascribable to an IMD (Table 3). One patient had presymptomatic metabolic testing for intrafamilial screening (previous case of IMD in the family: hereditary tyrosinemia type 1, HT1)^26^. Indeed, HT1 is rarely if ever symptomatic in the immediate neonatal period^27^.

Electroencephalogram and cerebral MRI were non-contributive tests. However, those tests are essential when seizures or neurologic deterioration occur especially regarding outcomes.

In our population, cardiomyopathies were detected especially in group 2 (Supplemental Table 1). Among them was a Fallot tetralogy, two hypertrophic cardiomyopathies associated with poorly controlled maternal diabetes mellitus, two myocardial failures occurring after a neonatal asphyxia and one congenital cardiomyopathy with cardiogenic shock. The sole case of metabolic cardiomyopathy was a dilated cardiomyopathy in a Long Chain 3-Hydroxyacyl-CoA Dehydrogenase (LCHAD) deficiency case. This emphasizes the importance of systematic metabolic investigation (plasma acylcarnitines and organic acids) in neonatal cardiomyopathies.

In groups 0, 2 and 3, abnormalities of metabolic testing were nonspecific especially for aminoacids and organic acids (except for CSF aminoacids). These nonspecific abnormalities were not checked when the clinical situation was not in the end attributed to an IMD or when another cause than IMD could be identified at the origin of the abnormality (e. g.: TPN and elevated aminoacids plasma levels; transient Krebs cycle intermediates in urine organic acids as already reported in healthy neonates^13^; milks containing medium chain triglycerides and abnormal organic acids^28^).

Such a diagnostic approach allowed survival and treatment of 4 neonates (maple syrup urine disease [MSUD], propionic acidemia [PA], carnitine-acylcarnitine translocase deficiency and HT1) (Table 9). Genetic counselling was possible for the further pregnancies in the families of the two fatal cases of urea cycle defects, allowing a prenatal diagnosis for one case and a presymptomatic treatment in the other. These two cases of urea cycle defect were treated with extracorporeal depuration and ammonia scavengers, but the treatment occurred too late to allow survival. Of note, although fully treatable with N-carbamylglutamate (NCGA), NAGS diagnosis was delayed and made when ammonia was already above 1000 µM and NCGA was tested too late. In total, 5 patients died in the neonatal period (one LCHAD deficiency, one N-acetyl-glutamate synthase (NAGS) deficiency, one type 1 citrullinemia, two non-ketotic hyperglycinemia) and 2 died later before 2 years of age (mitochondriopathies) (Table 9).

No OS or MPS analysis was ordered in group 1. In other groups, the results were mostly normal. Urine MPS/OS were performed in the setting of hepatosplenomegaly, fetal hydrops and/or hydramnios in association with abnormal neurological findings (in 29 patients, Fig. 2). Though we did not identify any mucopolysaccharidosis or oligosaccharidosis, such indications seem reasonable to propose for when to prescribe urinary MPS/OS.

The neurological signs in group 2 were due mainly to anoxo-ischemic encephalopathy (Supplemental Table 1), with lack of symptom-free interval and a suggestive perinatal context (low Apgar score) thus confirming the absence of IMD in this particular group (Table 6). The large amount of nonspecific abnormalities can question the detection threshold of “abnormal” metabolite levels in neonates^29^. Indeed, neonatal population in which those tests are prescribed is particular because neonates are sick, often hospitalised in NICU, receive TPN and drugs that can interfere with results of metabolic testing.

### Limitations and perspectives

Our study aimed at determining the diagnostic contribution of targeted metabolic testing in the diagnostic approach of IMD in neonates. It was not possible to calculate specificity or sensitivity of each metabolic testing because of the low number of positive cases and that all the included patients did not have the same metabolic testings (leaved to the choice of the clinician depending on the clinical scenario). However, the value of metabolic testing given specific clinical or laboratory findings is being emphasized in our study. For example, it is not surprising that 55% of all subjects with hyperammonemia (5/9) had an IMD nor is it surprising that only 7% of subjects with hypoglycemia had an IMD (2/30).

Importantly, it would be key to get information (if possible prospective) regarding long-term outcomes of patients from group 3, which indeed represents a “basket” with possibly unidentified genetic diseases. Next generation sequencing (NGS, whole exome and/or whole genome sequencing) would for sure be of interest in such patients^4,30–32^ but is not routinely available in our hospital^33–35^. Finally and most importantly, this study was performed in a country where expanded NBS for IMDs is in 2019 not yet available except for PKU.

### Screening of at-risk newborns versus general population

Our data provide an opportunity to infer whether NBS would have been advantageous in allowing to diagnosing IMD and further implement presymptomatic treatment. Although a larger population study would have been necessary in order to precisely address the screening versus symptomatic testing with numbers, it is important to note that while NBS would have theoretically allowed to diagnosing 6 of the IMDs: CACT, LCHAD, ASS, MSUD, PA and HT1 (provided succinylacetone was on the panel), severe clinical deterioration occurred before day 7 for 3 individuals that is before the results of NBS would have been available. For MSUD and PA, though clinical deterioration occurred slightly later at day 14 and day 12 of life respectively, it is also unlikely that a NBS approach would have prevented such clinical deterioration through presymptomatic management. Regarding, the 5 individuals who exhibited early death (LCHAD, NAGS, ASS, 2 NKH), NBS would not have been helpful: very early onset (first days of life) of the presentations as discussed above for LCHAD and ASS and no screen available for NAGS and NKH.

Last, the yield of metabolic testing has been established for other patient groups than newborns especially in selected groups such as neurodevelopmental disorders where it was shown that the contribution of systematic metabolic testing is up to 3%^36^. As stated by others, early identification of IMDs is key in order to flag treatable conditions^37^. Similar to non-targeted approaches such as NBS and additional genetic/metabolic studies^38^, an algorithm for selecting the patients in whom testing is performed is a useful tool. In our daily practice, this targeted approach based on symptoms suggestive of an IMD seems appropriate for the diagnosis of IMDs in the neonatal period.

## Conclusion

Non-systematic targeted metabolic workup in the neonatal population is based on clinical and laboratory parameters suspicion corresponding to a predefined clinical and laboratory parameters pattern. As opposed to NBS that targets presymptomatic (or asymptomatic) neonates, this diagnostic approach mainly concerns symptomatic neonates. In our study population, it is unlikely that a NBS approach (versus symptomatic testing) would have been advantageous due to the very early onset of the clinical presentations in the IMD individuals. The rate of 5.6% of IMD neonates in a selected population is rather satisfactory. Moreover, some true IMD neonates might have been missed (some of the group 3 individuals) and this is where other diagnostic tools (NBS and/or NGS) should deserve discussion. It could be possible that this diagnostic yield of IMD would have been higher if NBS for IMD had been available. Conversely, a rate of 23% healthy neonates metabolically tested seems acceptable for three reasons: (i) it allows a quick diagnosis of IMD that can be treatable, with better outcome, (ii) it limits the number of patients who die without any diagnosis been made provided the time to diagnostic and therapy is reduced, (iii) it allows prenatal diagnosis with genetic counselling for future pregnancies. Lastly, the cost-effectiveness of our approach is probably rather low.

## Supplementary information

Supplemental Table 1
