# Supplementary material for: Diagnostic contribution of metabolic workup for neonatal inherited metabolic disorders in the absence of expanded newborn screening
Source: Sci Rep. 2019 Oct 1;9:14098. doi: 10.1038/s41598-019-50518-0 (PMC6773867; doi:10.1038/s41598-019-50518-0)
Supplement: Supplementary file 1 — Supplemental Table 1 [file 41598_2019_50518_MOESM1_ESM.docx]

**Diagnostic contribution of metabolic workup for neonatal inherited metabolic disorders in the absence of expanded newborn screening**

Alexandra Bower^1,2^, Apolline Imbard^3,4^, Jean-François Benoist^3,4^, Samia Pichard^2^, Odile Rigal^3^, Olivier Baud^1,5^ and Manuel Schiff ^2,5*^

^1^Neonatal intensive care department, Robert Debré University Hospital, APHP, Paris, 75019, France

^2^Reference Center for Inborn Errors of Metabolism, Robert Debré University Hospital, APHP, Paris, 75019, France

^3^Biochemistry Laboratory, Robert Debré University Hospital, APHP, Paris, France

^4^Paris Sud University, Chatenay Malabry, France

^5^UMR1141, PROTECT, INSERM, Université Paris Diderot, Sorbonne Paris Cité, Paris, 75019, France

*Correspondence: Manuel Schiff, [manuel.schiff@aphp.fr](mailto:manuel.schiff@aphp.fr)

Supplemental Table 1: Diagnoses in group 2

| **Infectious diseases**  -CMV infection  -enterovirus infection  -E.Coli pyelonephritis  -Staphylococus pyogenes infection  -meningoencephalitis without bacterial documentation | **N=8**  N=2  N=3  N=1  N=1  N=1 |
| --- | --- |
| **Genetic disorder**  -Prader Willi syndrome  -Ondine syndrome  -KCNQ2 mutation  -BRAF mutation  -FGFR2 mutation (Crouzon disease)  -Chromosome 18 cytogenetic abnormality  -Chromosome 13 cytogenetic abnormality  -mutation in PTPN11 gene (Noonan syndrome)  -Cystic fibrosis  -osteogenesis imperfecta  -trichothiodystrophy | **N=16**  N=5  N=2  N=1  N=1  N=1  N=1  N=1  N=1  N=1  N=1  N=1 |
| **Hypoxic-ischemic encephalopathy** | **N=33** |
| **Epileptic disease** | **N=6** |
| **Cerebro vascular accident** | **N=7** |
| **Hematological disorder**  -Hemorrhage  -Anemia due to ABO incompatibility | **N=2**  N=1  N=1 |
| **Cardiopathy** | **N=5** |
| **Transient neonatal hyperinsulinism** | **N=7** |
| **Fetal alcoholic syndrome** | **N=2** |
| **Hirschprung disease** | **N=1** |
| **Congenital myopathy** | **N=2** |
